# Supplementary material for: Plasma Lipid Profile Among Perimenopausal Latvian Women in Relation to Dietary Habits
Source: Nutrients. 2025 Jan 10;17(2):243. doi: 10.3390/nu17020243 (PMC11767470; doi:10.3390/nu17020243)
Supplement: Supplementary file 1 [file nutrients-17-00243-s001.zip › nutrients-3409837-supplementary.pdf]

Supplementary materials:

Questionnaire about participants' characteristics S1.

Participant ID: _________

I. General information

1.1. Age: __________ (whole years)

1.2. Weight: __________ (kg) **1.3. Height:** __________ (cm)

1. 4. Waist circumference: __________ (cm) **1.5. Hip circumference:** __________ (cm)

1.6. Your region of residence:

☐Riga ☐Pierīga ☐Zemgale

☐Vidzeme ☐Kurzeme ☐Latgale

1.7. Your educational qualifications (indicate the highest level attained):

☐basic education ☐ general secondary ☐ secondary special

☐ incomplete higher education ☐ highest

1.8. Your occupation:

☐ full-time paid employee ☐ part-time paid employee ☐ self-employed

☐ unemployed ☐ housewife

1.9. Your marital status:

☐ unmarried ☐married ☐ divorced

☐ in a civil marriage ☐widow

1.10. Children under the age of 18 living in the family:

☐ no children ☐one ☐two

☐three ☐four or more

II. Lifestyle and health

2.1. In the last 6 months, have you taken any medication or supplements?

☐no ☐yes

2.1.1. If you answered NO to question 2.1, please go to question 2.2; if YES, please specify which:

- for digestive disorders
- antibiotics
- against insomnia
- against pain, fever, inflammation
- against convulsions
- hormonal medications
- against high blood pressure
- hormonal contraception
- hyperlipidaemic drugs
- medications to lower blood glucose levels
- vitamins, food supplements
- other: ____

2.1.2. Please indicate the names of taken medications:

____

2.2. Do you usually do light physical activity every day?
For example, light walking, moving around without a car, climbing stairs, and house cleaning.

- yes
- no
- more often yes
- more often no

2.3. How many minutes a week do you usually do moderate-intensity physical activity?
For example, brisk walking, swimming, cycling, gardening

- up to 150 minutes
- 150-300 minutes
- more than 300 minutes

2.4. How many minutes per week do you usually do high-intensity aerobic exercise?
For example, jogging, hard gardening (shovelling snow, digging up the garden), hiking in mountainous terrain

- up to 75 minutes
- 75-150 minutes
- more than 150 minutes

2.5. How many minutes per week do you usually do muscle-strengthening exercises for the main muscle groups?
For example, lifting weights, resistance exercises (pull-ups, push-ups, squats), and yoga.

- up to 2 times a week
- 2 times a week
- more than 2 times a week

2.6. Do you smoke?
Cigarettes, electronic cigarettes (number of cigarettes per day corresponds to the number of times per day you smoke)

- no, I've never smoked
- I don't smoke now, but I used to
- yes, but not every day
- yes, daily 1-5 cigarettes per day
- yes, at least 6-10 cigarettes every day
- yes, between 11 and one pack per day
- yes, more than one pack per day

II. Eating habits

3.1. What is your average daily number of meals (excluding snacks)?

- less than 2
- 2-3
- 4-5
- more than 5

3.2. What is your average number of snacks per day?

- no
- 1
- 2
- 3
- more than 4

3.3. Please indicate whether you have made any of the following changes to what you eat or drink in the last 6 months.

- I haven't changed anything
- smaller portions
- larger portions
- less fruit and vegetables
- more fruit and vegetables
- less meat
- more meat
- less sugar
- more sugar
- less alcohol
- more alcohol
- less drinking water
- more drinking water
- less salt
- more salt
- less fish
- more fish
- less sour milk products
- more sour milk products
- intermittent fasting

3.4. Do you exclude any foods from your daily diet (for health or other reasons):

- no
- fish
- seafood
- olas
- cow's milk
- lactose
- vegetables
- fruits, berries
- dried fruits
- potatoes
- nuts, seeds
- sour milk products
- cereals and their products
- gluten (wheat, barley, rye)
- poultry meat (chicken, turkey, duck, goose)
- animal meat (cattle, pigs, sheep, goats, rabbits)
- other: ________________________________

3.5. How often do you drink alcoholic beverages?

- never
- once a month or less
- 2-4 times a month
- once per week
- 2-3 times a week
- 4 times a week and more

3.5.1. If you answered NEVER to question 3.5, please go to question 4.1; if YES, please specify:
How much alcohol do you tend to consume on a typical drinking occasion?
One serving of alcohol:
beer 500ml - 1.6 servings; cocktail, cider 300ml - 1 serving; wine 150ml (glass) - 1.2 servings; spirits 200ml - 5 servings

☐ 1 or 2 servings ☐ 3 or 4 servings ☐ 5 or 6 servings

☐ 7 or 8 servings ☐ 10 servings or more

III. History of diseases

4.1 Have you ever been diagnosed with any of the following diseases?

Disease	No	Yes
**Hypertension** (high blood pressure)		
**Diseases of the digestive system** (stomach ulcers, gastritis, gastroesophageal reflux disease, irritable bowel syndrome)		
Other chronic diseases (if YES, please specify which)		

Other chronic diseases:

____

____

Table S1. Categories of food products and beverages.

Category	Examples of products
Grains, grain-based products	Pasta, rice, rice, buckwheat, breakfast porridges, barley, pearls, millet, amaranth, quinoa, sweet breakfast cereals, muesli
Bread	Rye bread, white bread, seed bread, galetes, bread rusks
Potatoes	Boiled, fried, French fries, potato pancakes
Meat, poultry	Pork, beef, veal, lamb, mutton, game meat (venison, wild boar, elk), rabbit meat, poultry
Meat offal	Liver, tongue, lungs, hearts, stomachs, salted bacon, smoked bacon
Meat products	Meat preserves, sausages, frankfurters, jamon/prosciutto, smoked meat
Eggs	Hen eggs, quail eggs
Fish	Freshwater fish, seawater fish
Fish products	Sprats, fish roe, fish sticks, crab sticks, seafood, cod liver
Milk, milk products	Milk, yoghurt, kefir, buttermilk, ayran, sour cream, cottage cheese, cheese, etc.
Vegetable oils, plant- and animal-based fats, dressings	Margarine, butter, olive oil, vegetable oils, seed oils, soy sauce, spicy sauces, white salad dressings, mayonnaise, tomato sauces, ketchup, etc.
Substitutes for meat products (plant-based)	Tofu, tempeh, soy meat
Dairy substitutes (plant-based)	Plant-based milk, plant-based cheese
Vegetables	Fresh, cooked, fermeted, canned vegetables
Fruits, berries	Fresh, canned and dried fruits and berries
Mushrooms	Wild and cultivated edible mushrooms
Legumes	Green beans, peas, lentils, chickpeas
Nuts, seeds	Walnuts, hazelnuts, peanuts, almonds, cashews, nut or seed butter/paste, flax seeds, chia seeds, sesame seeds, hemp seeds, sunflower seeds, pumpkin seeds, etc.
Sweets	Cookies, pastries, muffins, waffles, cakes, chocolate, halva, kozinaki, sherbet, cereal bars, candies, marshmallows, marmalade, fruit jelly, sorbet, ice cream, sweet popcorn, corn sticks, breadsticks
Sugars and related food	Jam, preserves, chocolate spread, sugar substitutes, sugar, syrups (agave, maple, etc.), honey
Fast food, salty snacks	Dumplings, meat pie, vegetarian pie, sausage roll, hot dog, cheburek, pizza, burger, tortilla, kebab, salted popcorn, crackers, chips

Lemonades, energy drinks	Lemonades, kvass, malt drinks, energy drinks

**Category**	**Examples of products**
Juices	Fruit juice 100%, fruit juice drinks, fruit juice nectar, vegetable and root juice
Alcoholic beverages	Wine, sparkling wine/prosecco, beer, sweet alcoholic drinks, alcohol drinks without sugar, non-alcoholic drinks (wine, sparkling wine, vermouth, gin, etc.)
Coffee, tea	Coffee, cocoa, chocolate drinks, instant coffee drink "3-in-1", latte, cappuccino, black or green tea, herbal tea
Water	Still water, carbonated mineral water with flavouring
Dietary supplements	B group vitamins, Omega-3, iron, magnesium, phospholipids, calcium, vitamin D, vitamin C, probiotics, fibre supplements, meal replacements, etc.
